# Impact of oral hygiene involving toothbrushing *versus* chlorhexidine in the prevention of ventilator-associated pneumonia: a randomized study

**DOI:** 10.1186/s12879-017-2188-0

**Published:** 2017-01-31

**Authors:** Claudia Fernanda de Lacerda Vidal, Aurora Karla de Lacerda Vidal, José Gildo de Moura Monteiro, Aracele Cavalcanti, Ana Paula Trindade Henriques, Márcia Oliveira, Michele Godoy, Mirella Coutinho, Pollyanna Dutra Sobral, Claudia Ângela Vilela, Bárbara Gomes, Marta Amorim Leandro, Ulisses Montarroyos, Ricardo de Alencar Ximenes, Heloísa Ramos Lacerda

**Affiliations:** 1 Tropical Medicine Health Sciences Center, Committee on Infection Control of Hospital das Clinicas, Universidade Federal de Pernambuco, Av. Professor Moraes Rego, 1235 Hospital das Clínicas - Cidade Universitária, Recife, Pernambuco 50670-901 Brazil; 2 Department of Pathology, Institute of Biological Sciences, Universidade de Pernambuco, Hospital de Câncer de Pernambuco, Real Hospital Português de Beneficência em Pernambuco, Recife, Pernambuco Brazil; 3 Cardiac Intensive Care Unit, Pronto-Socorro Cardiológico de Pernambuco, Universidade de Pernambuco, Recife, Pernambuco Brazil; 4 Committee on Infection Control, Pronto-Socorro Cardiológico de Pernambuco, Universidade de Pernambuco, Recife, Pernambuco Brazil; 5 Committee on Infection Control, Real Hospital Português de Beneficência em Pernambuco, Recife, Pernambuco Brazil; 6 Intensive Care Unit, Hospital Agamenon Magalhães, Secretaria de Saúde de Pernambuco, Recife, Pernambuco Brazil; 7 Intensive Care Unit, Hospital das Clínicas, Universidade Federal de Pernambuco, Recife, Pernambuco Brazil; 8 Committee on Infection Control of Hospital das Clinicas, Universidade Federal de Pernambuco, Recife, Pernambuco Brazil; 9 Institute of Biological Sciences, Universidade de Pernambuco, Recife, Pernambuco Brazil; 10 Faculty of Medical Sciences, Tropical Medicine Health Sciences Center, Universidade Federal de Pernambuco, Recife, Pernambuco Brazil; 11 Department of Infectious and Parasitic Diseases, Faculty of Medical Sciences, Tropical Medicine Health Sciences Center, Universidade Federal de Pernambuco, Recife, Pernambuco Brazil

**Keywords:** Pneumonia, Mechanical ventilator, Oral hygiene, Toothbrushing, Chlorhexidine, Intensive care

## Abstract

**Background:**

Nosocomial pneumonia has correlated to dental plaque and to oropharynx colonization in patients receiving mechanical ventilation. The interruption of this process, by preventing colonization of pathogenic bacteria, represents a potential procedure for the prevention of ventilator-associated pneumonia (VAP).

**Methods:**

The study design was a prospective, randomized trial to verify if oral hygiene through toothbrushing plus chlorhexidine in gel at 0.12% reduces the incidence of ventilatior-associated pneumonia, the duration of mechanical ventilation, the length of hospital stay and the mortality rate in ICUs, when compared to oral hygiene only with chlorhexidine, solution of 0.12%, without toothbrushing, in adult individuals under mechanical ventilation, hospitalized in Clinical/Surgical and Cardiology Intensive Care Units (ICU). The study protocol was approved by the Ethical Committee of Research of the Health Sciences Center of the Federal University of Pernambuco – Certificate of Ethical Committee Approval (CAAE) 04300012500005208. Because it was a randomized trial, the research used CONSORT 2010 checklist criteria.

**Results:**

Seven hundred sixteen patients were admitted into the ICU; 219 fulfilled the criteria for inclusion and 213 patients were included; 108 were randomized to control group and 105 to intervention group. Toothbrushing plus 0.12% chlorhexidine gel demonstrated a lower incidence of VAP throughout the follow up period, although the difference was not statistically significant (*p* = 0.084). There was a significant reduction of the mean time of mechanical ventilation in the toothbrushing group (*p* = 0.018). Regarding the length of hospital stay in the ICU and mortality rates, the difference was not statistically significant (*p* = 0.064).

**Conclusions:**

The results obtained showed that, among patients undergoing toothbrushing there was a significant reduction in duration of mechanical ventilation, and a tendency to reduce the incidence of VAP and length of ICU stay, although without statistical significance.

**Trial registration:**

Retrospectively registered in the Brazilian Clinical Trials Registry (Registro Brasileiro de Ensaios Clínicos) - RBR-4TWH4M (4 September 2016).

## Background

Nearly 9% to 40% of infections acquired in the Intensive Care Unit (ICU) are ventilator-associated pneumonia (VAP), and are related to increased length of hospital stay, higher morbidity and mortality, which significantly affects hospital costs [1, 2].

Nosocomial pneumonia has been correlated to dental plaque and to oropharynx colonization in patients receiving mechanical ventilation (MV). The endotracheal tube works as a conductor of the microorganisms of the oropharynx to the lower respiratory tract, and these are frequently identified as etiological agents of the nosocomial pneumonia [3–5].

The interruption of this process, by preventing colonization of pathogenic bacteria, represents a potential procedure for the prevention of VAP [6].

Considering that the microbiota of the oral cavity plays an important role in the development process of VAP, some studies have indicated that the topical application of chlorhexidine, initiated before intubation, reduces nosocomial infections in patients submitted to elective cardiac surgery [7, 8].

However, although the pharmacological control of bacterial plaque, through the use of chlorhexidine is practical and widely accepted among health professionals, the chemical approach against accumulated plaque is marginal, since the plaque acts as a biofilm in which the bacteria is considerably less sensitive to antimicrobial therapy – when compared to the free-moving planktonic form [9]. Therefore, mechanical cleansing, through toothbrushing may be the most effective method of removing all pathogens from the plaque, including anaerobes and multiresistant bacteria such as methicilline-resistant *Staphylococcus aureus*, (MRSA) or *Pseudomonas* [10].

The mechanical removal of microorganisms can increase the efficacy of the effects of chlorhexidine in the remaining bacteria or in bacterial regrowth, according to Kishimoto and Urade [11].

Although many studies suggest a potential relation between deficient oral care and increased incidence of VAP, the available evidence is still limited. This study was designed to verify if oral hygiene through toothbrushing with chlorhexidine in gel at 0.12% reduces the incidence of ventilatior-associated pneumonia, the duration of mechanical ventilation, the length of hospital stay and the mortality rate in ICU, when compared to oral hygiene only with chlorhexidine, solution of 0.12%, without toothbrushing, in adult individuals under mechanical ventilation, hospitalized in Clinical/Surgical and Cardiology Intensive Care Units. The toothbrushing is the basis for the removal of dental plaque and consequently reduction of oral bacterial load, reducing the risk for VAP.

## Methods

We conducted a prospective, randomized study of oral hygiene with 0.12% chlorhexidine solution every 12 h (control group) *versus* toothbrushing plus 0.12% chlorhexidine gel every 12 h (intervention group) in three ICU of public hospitals and one ICU of a philanthropic hospital in Recife, Brazil, from July 2013 to January 2014. The study protocol was approved by the Ethical Committee of Research of the Health Sciences Center of the Federal University of Pernambuco – CAAE 04300012500005208 and a written informed consent was obtained from all patients or relative before randomization. A research team was responsible for designing and executing the study, analyzing data, interpreting findings and writing the manuscript. The authors vouch for the accuracy and completeness of the reported data.

The primary endpoint was to assess the impact of introducing toothbrushing as a component of oral care on the incidence of VAP. The secondary endpoints were to identify differences in duration of mechanical ventilation, length of hospital stay and mortality rate in ICU between the studied groups.

### Recruitment, randomization and follow up

#### Study population

Individuals who were consecutive admitted into the four participating Intensive Care Units (total of 46 beds) and that fulfilled the inclusion criteria: age equal or greater than 18 years; submitted to intubation; expected to remain on mechanical ventilation for >48 h; and without evidence of pulmonary infection at admission. Individuals without teeth, suspicion of pneumonia at the time of intubation, pregnancy, tracheostomy and chlorhexidine allergy also were excluded.

The participants also underwent the standard protocol for prevention of VAP, which included maintaining a semirecumbent body position, with head elevation of ≥ 30°, gastrointestinal bleeding prophylaxis, deep venous thrombosis prophylaxis and daily interruption of sedation with assessing the possibility of extubation.

Among the four Intensive Care Units, three were medical/surgical with total of 36 beds and the other a cardiac ICU with 10 beds. About 65% of hospitalized patients in the ICU were medical care while 35% were surgical patients, and among medical patients, 20% were cardiac.

### Randomization

Patients were randomized within 24 h of intubation and initiation of mechanical ventilation for the control group (oral hygiene with 0.12% chlorhexidine solution every 12 h), or the intervention group (toothbrushing plus 0.12% chlorhexidine gel every 12 h) by means of opaque sealed envelopes containing the results from a computer generated random list.

Nurses responsible for assistance in ICU, previously trained by the research team, opened the envelope containing the assigned group within 24 h of intubation and included in the nursing systematized assistance plan the group of oral hygiene for which the patient had been randomized (control group or intervention group). Researchers and physicians did not know to which of both groups the individuals belonged, providing information to blind. The nurses and practical nurses were trained to implement oral hygiene according to the protocols established for both groups.

### Treatment regimens

Whereas in most of the studies reviewed, the concentration was more widely used chlorhexidine 0.12%, especially in the few studies comparing with toothbrushing, and the fact of the hospitals participating in the study had only the formulation of chlorhexidine 0.2% for use in the study population and the "kit" for toothbrushing, obtained through donations and own resources of the principal investigator, containing the antiseptic chlorhexidine in concentration 0.12%, the research team defined the use of two study groups, as described below.

### Control group

Individuals undergoing oral hygiene every 12 h, through aspiration of oropharyngeal secretion, immediately applying 15 ml of 0.12% chlorhexidine gluconate oral solution using a *swab* on all tooth surfaces, tongue and mucosal surface of the mouth. The whole process was performed by nursing staff and followed the specific standard operating procedure.

### Intervention group

Individuals undergoing oral hygiene every 12 h through aspiration of oropharyngeal secretion. Immediately after, toothbrushing was carried out on all tooth surfaces, tongue and mucosal surface of the mouth through the use of toothbrushes with small and soft bristles, and dental gel based on 0.12% chlorhexidine gluconate. After the previous steps proceeded with rinsing and suction through a catheter coupled to own toothbrush for this purpose aspiration. The whole process was performed by nursing staff and followed the specific standard operating procedure.

### Clinical dental examination

The Decayed, Missing and Filled Teeth Index (DMF) [12] was also calculated through the oral clinical exam, following the sequence of admission of the individuals in the study, by using a spatula and flat dental mirror under the light of unit with the examiner the patient’s right. Different dental spaces were examined one by one, systematically, including the right and left upper quadrants, and immediately the right and left lower quadrants.

### Definitions and data collection

Trainings were conducted by the principal investigator and by a collaborator dentist, to the whole team and all health professionals involved at the four participating institutions, with the aim of standardizing processes to operationalize the study, uniformity of approaches and calibration between participating professionals. This first stage of the study took place from July 2012 to July 2013, which enabled the start of randomization and data collection between July 2013 and January 2014.

After randomization, demographic, clinical and microbiological data was collected by the researchers throughout the follow up period of the individuals.

Based on clinical criteria, suspected VAP was defined as the presence of a new or progressive pulmonary infiltrate on chest radiography, associated to a minimum of two among three clinical criteria: fever (axilar temperature ≥37.8 °C), leukocytosis (>10 X 10^3^/mm^3^) or leukopenia (<3 X 10 X 10^3^/mm^3^), and purulent respiratory secretions (American Thoracic Society, 2005) – considering that bronchoscopy with quantitative cultures are not routinely used in the ICU study participants. Pneumonia defined by microbiological criteria included bacterial growth of endotracheal aspirates and bronchoalveolar lavage (bronchoscopic) with values ≥ 10^6^ cfu / ml and ≥ 10^4^ cfu / ml, respectively, associated with clinical criteria of pneumonia described above [13].

The clinical follow up included daily evaluation of the following data: temperature, leukocyte count, PaO_2_/FiO_2_ ratio, presence or absence of purulent respiratory secretions. Results of chest radiographies were routinely evaluated, as well as microbiological exams when available.

Early VAP defined as ventilator-associated pneumonia that occurs within four days of intubation whereas late-onset VAP as ventilator-associated pneumonia that occurs from the fifth day of intubation [13].

The participation of individuals ended on the 28th day of follow up or upon the occurrence of death, extubation or transfer.

### Statistical analysis

The sample size required to achieve a 50% reduction in suspected VAP, based on a VAP rate of 15,8% in the control group, with an 80% power and a error of 5%, was calculated to be 286 patients in each group. VAP incidence was reported as percentage and the incidence density as episodes per 1,000 days of mechanical ventilation. Discrete variables expressed as counts and percentages, and continuous variables as means and standard deviation (SD). The Decayed, Missing and Filled Teeth Index, calculated by the ratio between the total number of permanent teeth that are decayed, missed or filled and the total number of individuals of the sample, expressed as absolute number.

For the clinical and demographic characteristics of patients, differences between groups were assessed using Chi-square test for categorical variables, and Student *t*-test for continuous variables. The associations were expressed as Relative Risk (RR) and *p* values with 95% confidence interval (CI). In the multivariate analysis, logistic regression was applied to adjust potential confusion factors. The significance level of all the analyses was defined as *p* < 0,05. STATA version 12.0 was the software used for the analysis.

## Results

**Fig. 1:**
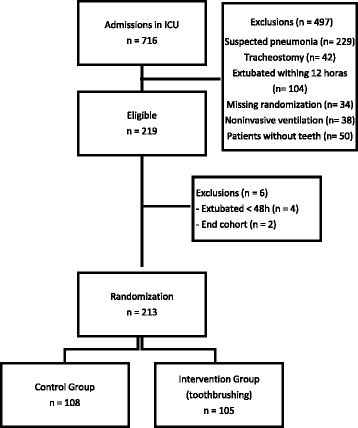
Diagram of patient inclusion in the study. Extubated <48 h = patients with mechanical ventilation expectancy longer than 48 h but extubated in the first 48 h extubation. End of cohort = patients with no definite outcome at the end of the study period

In the period from July 2013 to January 2014, were included 213 patients in the study, from which 108 were randomized to control group (oral hygiene with 0.12% chlorhexidine solution every 12 h) and 105 to intervention group (toothbrushing plus 0.12% chlorhexidine gel every 12 h. The patients were recruited from 4 Intensive Therapy Units in Recife, 69 patients (32.4%) being from Hospital 1, 50 patients (23.5%) from Hospital 2, 43 patients (20,2%) from Hospital 3, and 51 patients (23.9%) from Hospital 4. During this period, a total of 716 patients were admitted into the ICU of which 497 were excluded. Among the main causes of exclusion of patients admitted in ICU are suspected pneumonia admission, patients without teeth, tracheostomy, extubated withing 12 h wich resulted in failure to apply the oral hygiene protocol, missing randomization withing 24 h of admission. noninvasive ventilation. However, 219 fulfilled the criteria for inclusion in the study. Of these, 6 were later excluded; 4 had a mechanical ventilation period inferior to 48 h and 2 did not have defined outcomes due to the end of the study period (Fig. 1), in which resources are over.

Comparing the groups regarding clinical characteristics at admission, there was no statistically significant difference (*p* >0.05) (Table 1).

**Table 1:** Characteristics at ICU admission of patients who received oral hygiene with chlorhexidine 0.12% oral solution (control group) and toothbrushing with chlorhexidine gel 0.12% (intervention group)

Characteristics	Control group (*n* = 108)	Intervention group (*n* = 105)	*P* value
Sex			
Male	54 (50,0%)	51 (48,6%)	0,835
Female	54 (50,0%)	54 (51,4%)	
Age (in years)	63,2 ± 14,5	59,4 ± 14,5	0,059
Causes for intubation			
Acute respiratory failure secondary to pulmonary event	23 (21,3%)	29 (27,6%)	0,610
Acute respiratory failure secondary to cardiovascular event	51 (47,2%)	43 (40,9%)	
Acute respiratory failure secondary to neuromuscular event	6 (5,6%)	8 (7,6%)	
Acute respiratory failure secondary to foreing body aspiration	1 (0,9%)	0 (-)	
Other cause	27 (25,0%)	25 (23,8%)	
Intubation process			
Elective	23 (21,3%)	27 (25,7%)	0,678
Urgent	78 (72,2%)	70 (66,7%)	
Emergency	7 (6,5%)	8 (7,6%)	
Previous antibiotic use			
Yes	21 (19,4%)	26 (24,8%)	0,349
No	87 (80,6%)	79 (75,2%)	
APACHE II	22,2 ± 7,7	21,9 ± 7,5	0,767
Admission diagnosis			
Pulmonary disease	8 (18,6%)	5 (15,2%)	0,586
Cardiovascular disease	25 (58,1%)	23 (69,7%)	
Endocrine disease	2 (4,6%)	2 (6,1%)	
Cerebrovascular disease	0 (-)	1 (3,0%)	
Kidney disease	4 (9,3%)	1 (3,0%)	
Digestive disease	2 (4,6%)	1 (3,0%)	
Other	2 (4,6%)	0 (-)	
Comorbidities			
Pulmonary disease	25 (23,2%)	22 (21,0%)	0,699
Cardiovascular disease	92 (85,2%)	83 (79,1%)	0,242
Endocrine disease	66 (61,1%)	54 (51,4%)	0,154
Cerebrovascular disease	9 (8,3%)	11 (10,5%)	0,592
Kidney disease	22 (20,4%)	27 (25,7%)	0,354
Digestive disease	13 (12,0%)	17 (16,2%)	0,384
Hematologic disease	5 (4,6%)	4 (3,8%)	0,766

Among the 213 patients, ventilatior-associated pneumonia occurred in 45 (21.1%), 28 being patients from the control group and 17 from the intervention group, with incidence density equal to 14.2 by 1.000 MV/day. The use of toothbrushing plus 0.12% chlorhexidine gel demonstrated a lower incidence of VAP throughout the follow up period, although the difference was not statistically significant (*p* = 0.084). Clinical/radiological criteria defined 95.6% of cases of VAP; only 2 patients had microbiological diagnosis. Most cases of VAP (80%) occurred after the 4th day of mechanical ventilation (late-onset VAP). The relative risk of death was higher in the control group, increasing the risk of death by 41%, although it was not statistically significant (Table 2).

**Table 2:** Risk of VAP, duration of mechanical ventilation, length of stay and mortality in hospitalized patients in the ICU undergoing oral hygiene with chlorhexidine 0.12% oral solution (Control group) and toothbrushing with chlorhexidine gel 0.12% (Intervention group)

Events	Control group (*n* = 108)	Intervention group (*n* = 105)	RR	CI(95%)	*P* value
VAP					
No	80 (47,6%)	88 (52,4%)	1,0	-	-
Yes	28 (62,2%)	17 (37,8%)	1,81	0,93 – 3,57	0,084
Death					
No	81 (48,8%)	85 (51,2%)	1,0	-	-
Yes	27 (57,5%)	20 (42,5%)	1,41	0,73 – 2,70	0,296
Duration of mechanical ventilation^b^					
Mean ± sd	11,1 ± 7,6	8,7 ± 5,0	1,063	1,011 – 1,120	0,018^a^
Categorization^c^					
Up to 5 days	13 (37,1%)	22 (62,9%)	1,0	-	-
6 to 10 days	40 (48,8%)	42 (41,2%)	1,61	0,71 – 3,70	0,249
11 days and more	28 (57,1%)	21 (42,9%)	2,27	0,93 – 5,55	0,073
Length of ICU^b^					
Mean ± sd	13,9 ± 8,6	11,9 ± 7,77	1,032	0,999 – 1,065	0,064
Categorization^d^					
Up to 5 days	11 (39,3%)	17 (60,7%)	1,0	-	-
6 to 10 days	38 (50,0%)	38 (50,0%)	1,54	0,64 – 3,70	0,333
11 days and more	59 (54,1%)	50 (45,9%)	1,82	0,78 – 4,34	0,164

^a^statistically significant association

^b^Among patients who were discharged from the ICU (*n* = 166)

^c^Chi-squared test for trend (*χ* ^2^ = 3,205; *p* = 0,073)

^d^Chi-squared test for trend (*χ* ^2^ = 1,801; *p* = 0,179)

When considering the patients who were discharged from the ICU, there was a significant reduction of the mean time of mechanical ventilation in the group of patients who were submitted to toothbrushing (*p* = 0.018). The categorized analysis on duration of mechanical ventilation, there was a tendency for increased risk of long stay in mechanical ventilation for the control group (Chi-square for trend *p* = 0.073) (Table 2).

Regarding the length of hospital stay in the ICU, the difference was not statistically significant (*p* = 0.064), but there was a tendency to reduce in the length of stay in the ICU for the intervention group (Table 2).

Overall, the results showed a better scenario among patients undergoing toothbrushing. However, regarding the risk of VAP and death, the sample seems insufficient in size to detect a difference.

With respect to oral health status of the population, after stratification of the sample according to age, the DMF was of 24.9, 25.6, 26.4 and 27.0, for ages 45 to 54 years, 55 to 64 years, 65 to 74 years and 75 years of more, respectively. The amount of missing teeth accounted for more than 50% of the index in each of the age groups. Mean number of teeth present in the mouth, with respect to age groups, was 18.5 from 45 to 54 years; 14.8 from 55 to 64 years; 13.7 from 65 to 74 years and 10.3 for 75 years or more.

In the analysis of the clinical signs of periodontal disease, the most common findings were gingivitis and periodontitis, where 72% of the sample showed any sign of periodontal disorder characterized by the presence of tartar; reddened, swollen and bleeding gums; gingival pockets; gingival recession and tooth mobility.

Finally, no adverse events were reported associated with toothbrushing or chlorhexidine use.

## Discussion

In the present study, the use of toothbrushing plus 0,12% chlorhexidine gel demonstrated a lower incidence of VAP during the follow up period (28 VAP cases – control group X 17 VAP cases – intervention group), but the difference was not statistically significant (*p* = 0.084). Despite this, there was a significant reduction in the mean time of mechanical ventilation in the group of patients who were submitted to toothbrushing (*p* = 0.018). This study identified a tendency for shorter length of ICU stay and reducing mortality for the toothbrushing group, although without statistical significance. However, there was an increase of 41% in the relative risk of death for the control group, which reinforces the trend toward better clinical outcome for the intervention group.

There are many studies designed to prove the role of mechanical cleansing of dental plaque and its association with the reduction of VAP [14–16], but the results are limited.

Systematic revision and meta-analysis, including four studies with a total of 828 patients submitted to oral hygiene with and without toothbrushing, did not demonstrate benefits regarding reduction of VAP, duration of MV or length stay in ICU, for the toothbrushing group [17].

Alhazzani et al. [18] recently published a systemic revision and meta-analysis, to formulate a critical analysis of the impact of using toothbrushing as part of oral hygiene for individuals under intensive care and mechanical ventilation – analyzing studies published between 1980 and March of 2012. Six randomized studies, involving 1,408 patients – from which five compared toothbrushing with standard oral hygiene, and the sixth compared manual toothbrushing *versus* electric toothbrushing – fulfilled the inclusion criteria. Four studies demonstrated a tendency to lower rates of ventilator-associated pneumonia, although without statistical significance (*p* = 0.26). One only study, which presented low bias risk estimated by the Cochrane method, suggested that toothbrushing significantly reduced VAP occurrence (*p* = 0.006). No difference was observed between manual or electric toothbrushing. Moreover, there were no statistically significant differences regarding length of ICU stay or hospital mortality.

Although our study has demonstrated significant reduction in meantime mechanical ventilation, one limitation was the small sample size through interruption of the study.

The study discontinuation, due to lack of inputs for oral hygiene according to the study protocol, contributed to the failure to achieve the number of patients needed to more appropriate analysis of any difference between the groups. In fact, the sample calculation considered an VAP incidence in our region; however, financial resources ended, hospitals could not comply with the acquisition of materials needed to continue the search, and the data were analyzed with discontinuation of the study, what is meant a limitation of the research. Despite of this, like the studies above cited, tendency for lower incidence of VAP for the intervention group was found in our study.

Unfortunately, we could not work with the ideal number of participants, but even with these difficulties, the results showed trend to better outcomes with the intervention, and significant difference in mean time mechanical ventilation. Thus, operational difficulties relating to financial resources, considering the study in a country with few resources, developing country, and three public hospitals among 04 hospitals, which also involves little financial resources, defined the completion of research ahead of schedule time.

In spite of this, we consider relevant findings, which indicate a significant difference in mean time mechanical ventilation, and show a tendency to lower risk of death and lower absolute number of VAP in the population under the intervention.

Also it chose to work with only to study groups and use of chlorhexidine 0.12%, approved and widely used in various studies, which would allow comparison of the control group with the intervention group, to which the only difference is the use of brushing, since also in this group was the dental gel with 0.12% chlorhexidine. Thus, avoiding bias in relation to different concentrations of chlorhexidine and proceeds with the analysis of the differential component that has been the target of research, the role of brushing. The restriction on the number of treatment arms was importante, since the power would not have been suficiente.

In addition, the high VAP incidence (14.1/1,000 MV-day) pointed out in this study, when compared with data from the National Healthcare Safety Network (2.1 to 10.7 per 1,000 MV-day) [19], denotes the necessity to adopt more effective proven measures to reduce pneumonia in patients who are undergoing mechanical ventilation in our Intensive Care Units.

Although the four participating institutions of the study introduced the “bundle” for VAP prevention [20], the fifth component of the package of measures – oral hygiene – was not contemplated in the units before this study, and the justifications found for this gap point to technical difficulties, lack of knowledge about the importance of the measure by the professional team at the ICU, and lack of a standard protocol and adequate material resources. This gave rise to efforts, by the multiprofessional health team – made up of nurses, physicians, hospital infection control service professionals, physiotherapists and surgeon-dentist – in order to address the technical and material deficiencies, and effective application of a standard protocol for oral hygiene, including toothbrushing, for planning this randomized study.

In the last two decades, numerous published data has shown that inadequate oral hygiene increases the incidence of pneumonia both in the community and in hospitalized individuals undergoing intensive care [21]. Dental plaque serves as a reservoir for microorganisms associated to pulmonary infections, and these respiratory pathogens quickly colonize the plaque of patients hospitalized in ICU undergoing mechanical ventilation [3, 4]. Thus, care protocols represent an essential component for the reduction of VAP [21]. In order to control vies information in our study, a team of dentists performed the dental evaluation in all patients in the study, and have trained all nursing professionals to oral hygiene with toothbrushing with chlorhexidine 0.12%, through a standard protocol for oral care.

From that oral assessment, another finding was the high DMF index described in this study, despite the limitations of the indicator itself – obtained from the clinical examination restricted to the crown of the tooth and not showing secondary tooth losses to periodontal disease or orthodontic reasons [22] – denotes the level of oral health impairment for this population group. Measures are necessary to better promote oral health, since these critical patients who undergoing to mechanical ventilation present high risk of infection, especially of the inferior respiratory tract.

The following question remains: Why measure as effective for plaque rupture fails to demonstrate proven benefit in this patient population? The results of the different studies must be analyzed cautiously. First, establishing the VAP diagnosis for patients undergoing mechanical ventilation is much more complex when compared with community-acquired pneumonia. In addition, opinions among physicians about the diagnostic criteria also differ. Thus, the few studies included in the meta-analysis of Gu et al. [17], who used the VAP as the main outcome, could present disappointing results in relation to evidence about the expected superiority of toothbrushing as method of pneumonia prevention [21]. In our study, clinical/radiological criteria were used for the diagnosis of VAP, which could result in misdiagnosis, once the gold standard is represented by microbiological diagnosis. To minimize this possible bias in classification, standardization of clinical criteria, training of physicians responsible for diagnosing and *Kappa* test were used, which enabled the standardization of concepts and validate the diagnosis of VAP in this study. With the objective of minimizing possible bias of information and/or classification, different teams of professionals were defined to apply the oral hygiene protocol (nursing) and definition of VAP diagnosis (medical).

Another important point is that more precise criteria for investigation of the role of dental plaque rupture should be used when the design of studies which seeking to validate the role of toothbrushing as a primary measure for VAP prevention. The use of scores to evaluate dental plaque, suggested by Wise and Williams [21], helps to prove the efficacy of toothbrushing and makes possible the analysis of its influence in VAP incidence. Observational study demonstrated an increase in the occurrence of dental bacterial plaque along the length of intubation using dental plaque scores [23].

It is difficult to interpret the studies that do not show a reduction in VAP occurrence. The results of these studies could reflect mistakes during the toothbrushing procedure, that is, no reduction in the plaque score or removal of dental plaque by itself would not affect the incidence of VAP [21]. Moreover, the use of chlorhexidine appears to attenuate the effects of toothbrushing on VAP (*p* for interaction = 0.02) [18]. According to Labeau [24], the well-conducted meta-analysis by Alhazzani et al. [18] supports toothbrushing as a potential strategy for reducing VAP and oral care without the application of this method should be considered, at least, an improper practice.

Improving mouth hygiene represents one of innumerous interventions that can affect VAP occurrence [25]. The ideal would be to design more studies to define the adequate method for oral hygiene in this population of patients, using more precise measurements to validate the removal of dental plaque (plaque score) by toothbrushing, having as the main outcome the mortality rate. This would implicate a great number of recruits before the planning of studies evaluating VAP incidence, with greater probability of bias because of the diagnostic complexity, which is something that would complicate the interpretation of the results.

## Conclusions

In summary, the results obtained showed that, among patients undergoing toothbrushing there was a significant reduction in duration of mechanical ventilation, and a tendency to reduce the incidence of VAP and length of ICU stay, although this last results without statistical significance. Therefore, about the risk of VAP risk and death, the sample no appears to have been large enough to detect differences in this magnitude. More studies are needed in order to define optimal oral hygiene, use of dental plaque score, and observation of the impact of oral hygiene measures, mainly on hospital and ICU mortality rates.

## Abbreviations

CAAE: Certificate of ethical committee approval
CI: Confidence interval
DMF: Decayed, missing and filled teeth index
ICU: Intensive care unit
MRSA: Methiciline-resistant *staphylococcus aureus*
MV: Mechanical ventilator
RR: Relative risk
SD: Standard deviation
VAP: Ventilator associated pneumonia
