# Erratum to: Impact of oral hygiene involving toothbrushing versus chlorhexidine in the prevention of ventilator-associated pneumonia: a randomized study

**DOI:** 10.1186/s12879-017-2273-4

**Published:** 2017-02-27

**Authors:** Claudia Fernanda de Lacerda Vidal, Aurora Karla de Lacerda Vidal, José Gildo de Moura Monteiro, Aracele Cavalcanti, Ana Paula da Costa Henriques, Márcia Oliveira, Michele Godoy, Mirella Coutinho, Pollyanna Dutra Sobral, Claudia Ângela Vilela, Bárbara Gomes, Marta Amorim Leandro, Ulisses Montarroyos, Ricardo de Alencar Ximenes, Heloísa Ramos Lacerda

**Affiliations:** 1 Tropical Medicine Health Sciences Center, Committee on Infection Control of Hospital das Clinicas, Universidade Federal de Pernambuco, Av. Professor Moraes Rego, 1235 Hospital das Clínicas - Cidade Universitária, Recife, Pernambuco 50670-901 Brazil; 2 Department of Pathology, Institute of Biological Sciences, Universidade de Pernambuco, Hospital de Câncer de Pernambuco, Real Hospital Português de Beneficência em Pernambuco, Recife, Pernambuco Brazil; 3 Cardiac Intensive Care Unit, Pronto-Socorro Cardiológico de Pernambuco, Universidade de Pernambuco, Recife, Pernambuco Brazil; 4 Committee on Infection Control, Pronto-Socorro Cardiológico de Pernambuco, Universidade de Pernambuco, Recife, Pernambuco Brazil; 5 Committee on Infection Control, Real Hospital Português de Beneficência em Pernambuco, Recife, Pernambuco Brazil; 6 Intensive Care Unit, Hospital Agamenon Magalhães, Secretaria de Saúde de Pernambuco, Recife, Pernambuco Brazil; 7 Intensive Care Unit, Hospital das Clínicas, Universidade Federal de Pernambuco, Recife, Pernambuco Brazil; 8 Committee on Infection Control of Hospital das Clinicas, Universidade Federal de Pernambuco, Recife, Pernambuco Brazil; 9 Institute of Biological Sciences, Universidade de Pernambuco, Recife, Pernambuco Brazil; 10 Faculty of Medical Sciences, Tropical Medicine Health Sciences Center, Universidade Federal de Pernambuco, Recife, Pernambuco Brazil; 11 Department of Infectious and Parasitic Diseases, Faculty of Medical Sciences, Tropical Medicine Health Sciences Center, Recife, Pernambuco Brazil

## Erratum

In the version of this article that was originally published [1] there was an error with the author name “Ana Paula da Costa Henriques” since it was incorrectly listed as “Ana Paula Trindade Henriques”. The original article has been revised.
